# Impact of blood pressure control on retinal microvasculature in patients with chronic kidney disease

**DOI:** 10.1038/s41598-020-71251-z

**Published:** 2020-08-31

**Authors:** Shu-Yen Peng, Yih-Cherng Lee, I.-W.e n Wu, Chin-Chan Lee, Chi-Chin Sun, Jian-Jiun Ding, Chun-Fu Liu, Ling Yeung

**Affiliations:** 1 Department of Ophthalmology, Keelung Chang Gung Memorial Hospital, No. 222 Mai-Chin Road, Keelung, 204 Taiwan; 2 Graduate Institute of Communication Engineering, National Taiwan University, Taipei, Taiwan; 3 School of Medicine, College of Medicine, Chang Gung University, Taoyuan, Taiwan; 4 Department of Nephrology, Keelung Chang Gung Memorial Hospital, Keelung, Taiwan; 5 Community Medicine Research Center, Keelung Chang Gung Memorial Hospital, Keelung, Taiwan; 6 Department of Medical Research and Development, Chang Gung Memorial Hospital, Keelung, Taiwan; 7 Department of Electrical Engineering, National Taiwan University, Taipei, Taiwan; 8 Program in Molecular Medicine, National Yang Ming University, Taipei, Taiwan

**Keywords:** Chronic kidney disease, Circulation, Blood flow, Hypertension, Predictive markers

## Abstract

Chronic kidney disease (CKD) is an emerging disease worldwide. We investigated the relationship between blood pressure (BP) control and parafoveal retinal microvascular changes in patients with CKD. This case–control study enrolled 256 patients with CKD (stage 3–5) and 70 age‐matched healthy controls. Optical coherence tomography angiography showed lower superficial vascular plexus (SVP) vessel density, lower deep vascular plexus (DVP) vessel density, and larger SVP flow void area in the CKD group. The BP parameters at enrollment and during the year before enrollment were collected in patients with CKD. Partial correlation was used to determine the relationship between BP parameters and microvascular parameters after controlling for age, sex, diabetes mellitus, axial length, and intraocular pressure. The maximum systolic blood pressure (SBP) (p = 0.003) and within-patient standard deviation (SD) of SBP (p = 0.006) in 1 year were negatively correlated with SVP vessel density. The average SBP (p = 0.040), maximum SBP (p = 0.001), within-patient SD of SBP (p < 0.001) and proportion of high BP measurement (p = 0.011) in 1 year were positively correlated with the SVP flow void area. We concluded that long-term SBP was correlated with SVP microvascular injury in patients with CKD. Superficial retinal microvascular changes may be a potential biomarker for prior long-term BP control in these patients.

## Introduction

Chronic kidney disease (CKD) is an emerging disease worldwide that is highly prevalent in elderly individuals and in patients with hypertension or diabetes mellitus (DM)^1^. Approximately 10–15% of the world’s population have CKD^2^, and the prevalence could be as high as 40% among diabetic patients^3^. CKD is associated with many systemic vascular complications such as atherosclerosis, cerebrovascular disease, and cardiovascular disease^1^. End-stage kidney disease may increase the risk of cardiovascular mortality by 10–30 folds^1^.

Retinal vasculature, which can be directly visualized using non-invasive tools, has been considered a potential window for systemic vascular complications^4–6^. Fundus photography provided a convenient tool for correlating the risk of systemic vascular diseases and retinal vasculature [e.g. the central retinal arteriolar equivalent (CRAE) and the central retinal venular equivalent (CRVE)]^5^. However, recent publications showed that systemic diseases may cause retinal capillary alterations preceding any visible pathology in fundus^7–9^. Optical coherence tomography angiography (OCTA) technology provides a rapid non-invasive method to quantify microvasculature at the capillary level in the different retinal layers. It could detect early subtle retinal microvascular changes from systemic diseases^8–11^.

Using OCTA, we had demonstrated decreased parafoveal microvascular vessel densities in both superficial vascular plexus (SVP) and deep vascular plexus (DVP) in patients with CKD^12^. eGFR was an independent factor associated with SVP vessel density^12,13^. However, the pathogenesis of these microvascular rarefactions in patients with CKD is yet to be determined^2,12^. Hypertension commonly comorbid with CKD; more than 80% of patients with CKD may have hypertension^14^. Nevertheless, the effects of hypertension on different layers of the retinal microvasculature are controversial^10,15–17^. We hypothesized that poor blood pressure (BP) control in patients with CKD may result in various microvascular changes among different retinal layers. This study aimed to evaluate the relationship between different BP parameters and microvascular changes in different retinal layers in patients with CKD. This information could help guide the management of patients with CKD and preventing retinal microvascular injury in them.

## Results

In total, 256 patients with CKD and 70 healthy controls were enrolled in this study. The demographic data and clinical characteristics of the participants in both groups are summarized in Table 1. The mean age of the participants in the CKD and control groups was 62.4 ± 9.9 years and 63.0 ± 8.9 years, respectively. There was no statistical difference in age, sex, intraocular pressure, axial length, and foveal avascular zone size between the two groups. However, compared with controls, patients with CKD had worse best- corrected visual acuity (BCVA), lower SVP vessel density, lower DVP vessel density, and larger parafoveal SVP flow void area. Due to the wide range of age distribution, the standard deviations (SD) of SVP and DVP vessel densities were slightly higher than expected, but these values were comparable to recent large population-based study^18^.

**Table 1:** Demographic data and clinical characteristics of 326 participants.

	Control group (n = 70)	CKD group (n = 256)	*p* value
Age (mean ± SD)	63.0 ± 8.9	62.4 ± 9.9	0.645
**Sex, n (%)**			0.220
Female	35 (50)	107 (42)	
Male	35 (50)	149 (58)	
**Blood pressure, mmHg (mean ± SD)**			
SBP at enrollment	134 ± 16	136 ± 20	0.633
DBP at enrollment	75 ± 10	76 ± 13	0.899
Average SBP in 1 year	–	135 ± 16	
Average DBP in 1 year	–	76 ± 11	
Maximum SBP in 1 year	–	155 ± 23	
Maximum DBP in 1 year	–	86 ± 13	
Within-patient SD of SBP	–	14.0 ± 6.6	
Within-patient SD of DBP	–	7.3 ± 3.2	
LogMAR best corrected visual acuity (mean ± SD)	0.083 ± 0.117	0.122 ± 0.143	**0.021**
Intraocular pressure, mmHg (mean ± SD)	15.3 ± 2.4	15.1 ± 2.5	0.525
Axial length, mm (mean ± SD)	23.89 ± 0.9	23.72 ± 1.1	0.218
Parafoveal SVP vessel density, % (mean ± SD)	49.0 ± 3.7	46.9 ± 4.5	** < 0.001**
Parafoveal DVP vessel density, % (mean ± SD)	52.0 ± 3.1	50.9 ± 3.9	**0.034**
Parafoveal SVP flow void area, mm^2^ (mean ± SD)	0.142 ± 0.084	0.307 ± 0.236	** < 0.001**
Foveal avascular zone size, mm^2^ (mean ± SD)	0.312 ± 0.121	0.329 ± 0.121	0.299

Bold values indicate *p* < 0.05.

CKD: chronic kidney disease; DBP: diastolic blood pressure; DVP: deep vascular plexus; LogMAR: logarithm of the minimum angle of resolution; SBP: systolic blood pressure; SD: standard deviation; SVP: superficial vascular plexus.

The mean duration of CKD was 7.8 ± 6.0 years. The CKD group consisted of 114 patients (44.5%) with stage 3, 47 (18.4%) patients with stage 4, and 95 (37.1%) patients with stage 5 disease. In total, 40 (42%) and 38 (40%) patients with stage 5 CKD were undergoing hemodialysis and peritoneal dialysis, respectively. The mean serum creatinine (Cr) of the patients in the CKD group was 4.7 ± 4.4 mg/dL, mean blood urea nitrogen was 44.2 ± 27.8 mg/dL, and mean estimated glomerular filtration rate (eGFR) was 27.8 ± 20.3 mL/min/1.73 m^2^. In total, 111 (43%) patients had DM whereas 221 (86%) patients had a history of hypertension. Recent (within 3 months) glycosylated hemoglobin (HbA1c) level was available in 93 of the 111 CKD patients with DM. The mean HbA1c was 7.2 ± 1.8%. The mean duration of hypertension was 7.6 ± 5.2 years.

In this study, the BP of most patients with CKD was well controlled during enrollment. There were no statistical differences in systolic blood pressure (SBP) and diastolic blood pressure (DBP) at enrollment between the CKD and control groups (Table 1). Details of the average SBP/DBP, maximum SBP/DBP, and the within-patient SD of SBP/DBP in 1 year in the CKD group are shown in Table 1. BP of the patients was measured 8.7 ± 6.1 times in the year preceding this study. The proportion of high SBP measurement was ≤ 0.25 in 118 (46%) patients, 0.26–0.50 in 67 (26%) patients, 0.51–0.75 in 42 (16%) patients and > 0.75 in 29 (11%) patients, respectively. There was no significant difference between start and end SBP/DBP in this study (SBP mean difference − 1.6 mmHg, p = 0.316; DBP mean difference 0.4 mmHg, p = 0.645).

Table 2 shows the partial correlation between BP parameters and the parafoveal retinal microvascular parameters among patients with CKD after controlling for age, sex, DM, axial length, and intraocular pressure. Maximum SBP (p = 0.003) and within-patient SD of SBP (p = 0.006) were negatively correlated with SVP vessel density. Average SBP (p = 0.040), maximum SBP (p = 0.001), within-patient SD of SBP (p < 0.001) and proportion of high SBP measurement (p = 0.011) were positively correlated with parafoveal SVP flow void area. There was no significant correlation between BP parameters and DVP vessel density. DBP parameters, duration of CKD, and duration of hypertension had no significant correlation with any of the retinal microvascular parameters. Among patient with CKD and DM, the HbA1c level was correlated with SVP vessel density (− 0.328, p = 0.002), but not correlated with DVP vessel density (p = 0.479) and SVP flow void area (p = 0.103).

**Table 2:** Partial correlation between blood pressure parameters and parafoveal retinal microvascular parameters among 256 patients with CKD.

	Parafoveal SVP vessel density		Parafoveal DVP vessel density		Parafoveal SVP flow void area	
	Coefficient	P value*	Coefficient	P value*	Coefficient	P value*
SBP at enrollment	− 0.010	0.877	0.045	0.481	− 0.031	0.626
DBP at enrollment	0.038	0.554	0.053	0.402	− 0.100	0.115
Average SBP in 1 year	− 0.120	0.057	− 0.043	0.499	0.130	**0.040**
Average DBP in 1 year	− 0.013	0.841	0.084	0.185	0.004	0.950
Maximum SBP in 1 year	− 0.186	**0.003**	− 0.069	0.277	0.210	**0.001**
Maximum DBP in 1 year	− 0.069	0.277	0.052	0.414	0.068	0.285
Within-patient SD of SBP	− 0.174	**0.006**	− 0.071	0.265	0.244	** < 0.001**
Within-patient SD of DBP	− 0.086	0.176	− 0.022	0.724	0.095	0.132
Proportion of high SBP measurement	− 0.107	0.092	− 0.048	0.451	0.161	**0.011**

Bold values indicate *p* < 0.05.

CKD: chronic kidney disease; DBP: diastolic blood pressure; DVP: deep vascular plexus; SBP: systolic blood pressure; SD: standard deviation; SVP: superficial vascular plexus.

*p values were calculated by partial correlation after controlling for age, sex, diabetes mellitus, axial length, and intraocular pressure.

**Figure 1:**
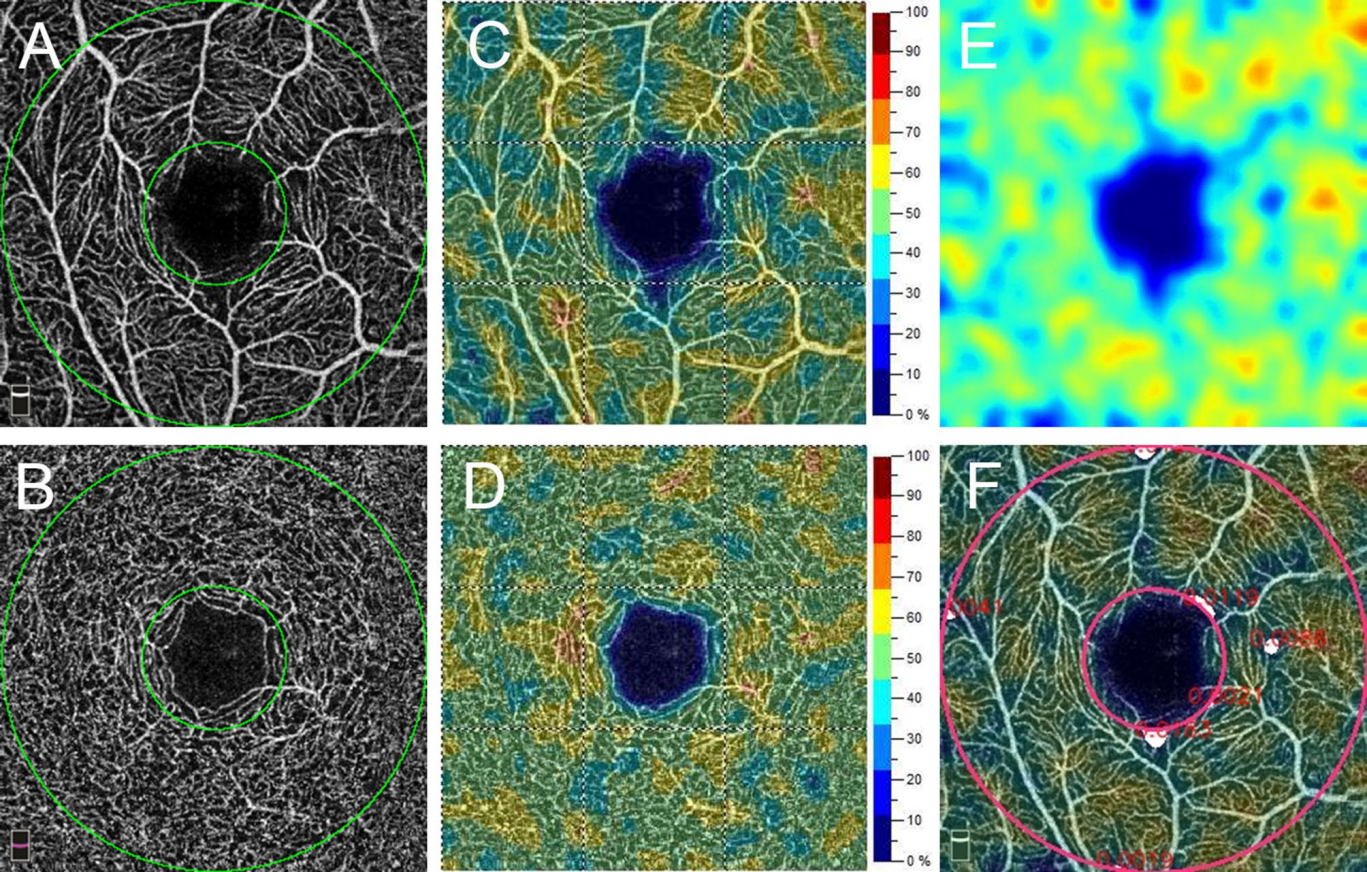
A 66-year-old man with chronic kidney disease (CKD) with normal average (131 mmHg) and maximum systolic blood pressure (132 mmHg) in one year. A 3 mm × 3 mm optical coherence tomography angiography (OCTA) image of (**A**) superficial vascular plexus (SVP) and (**B**) deep vascular plexus (DVP). Parafoveal area is the area between 2 green circles. The OCTA machine automatically calculated the vessel density map of (**C**) SVP and (**D**) DVP. (**E**) Vascular perfusion map of SVP was created from the algorism in this study. (**F**) The vascular perfusion map was superimposed on the SVP OCTA image. The parafoveal SVP flow void area (white color) was 0.06 mm^2^.

**Figure 2:**
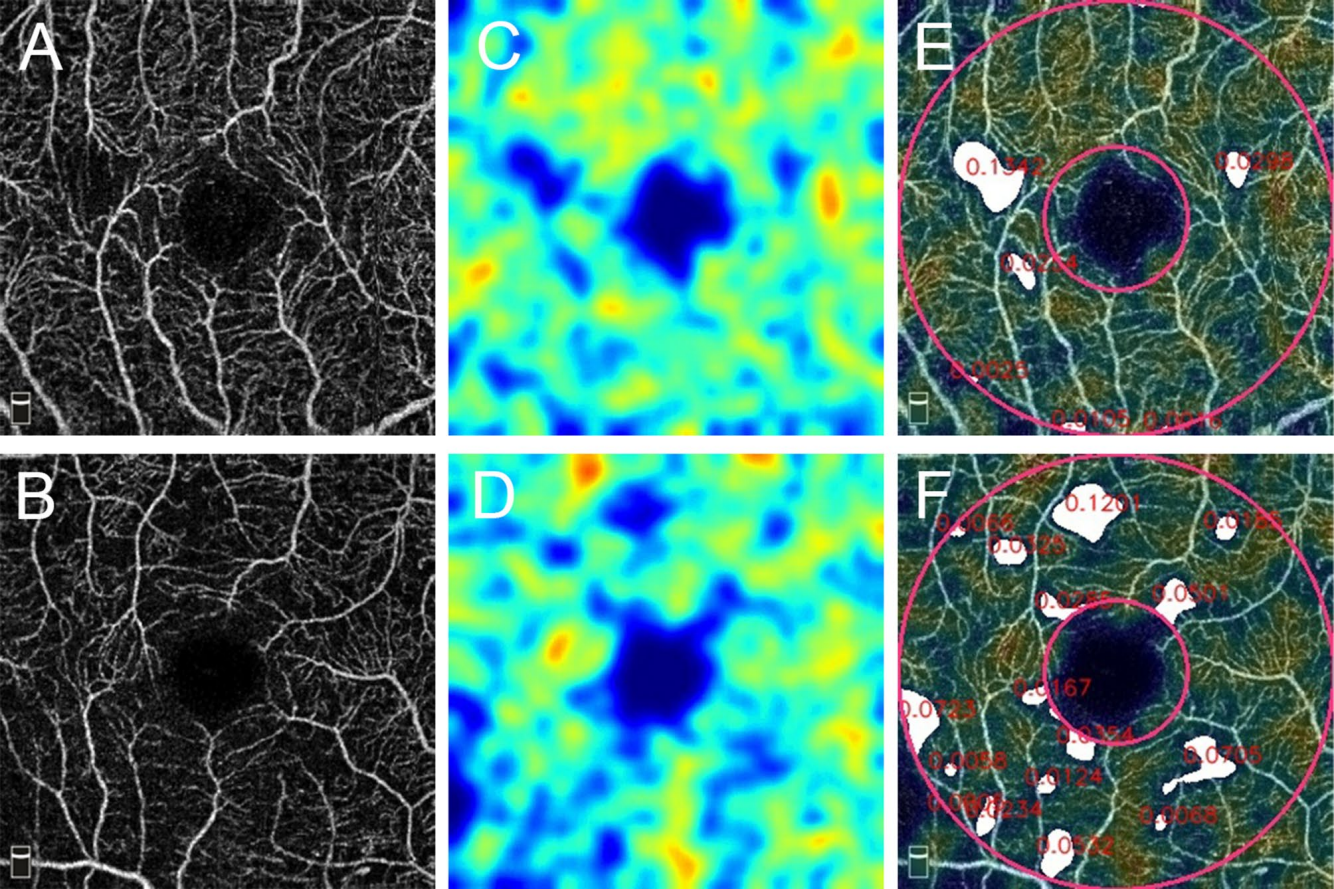
Two representative patients with high average systolic blood pressure (SBP) (146 mmHg and 158 mmHg) and maximum SBP (179 mmHg and 180 mmHg, respectively). (**A**,**B**) Optical coherence tomography angiography (OCTA) images of superficial vascular plexus (SVP). (**C**,**D**) Vascular perfusion map calculated from the algorithm in this study. (**E**,**F**) The parafoveal SVP flow void areas (white color) are 0.25 mm^2^ and 0.55 mm^2^, respectively.

**Figure 3:**
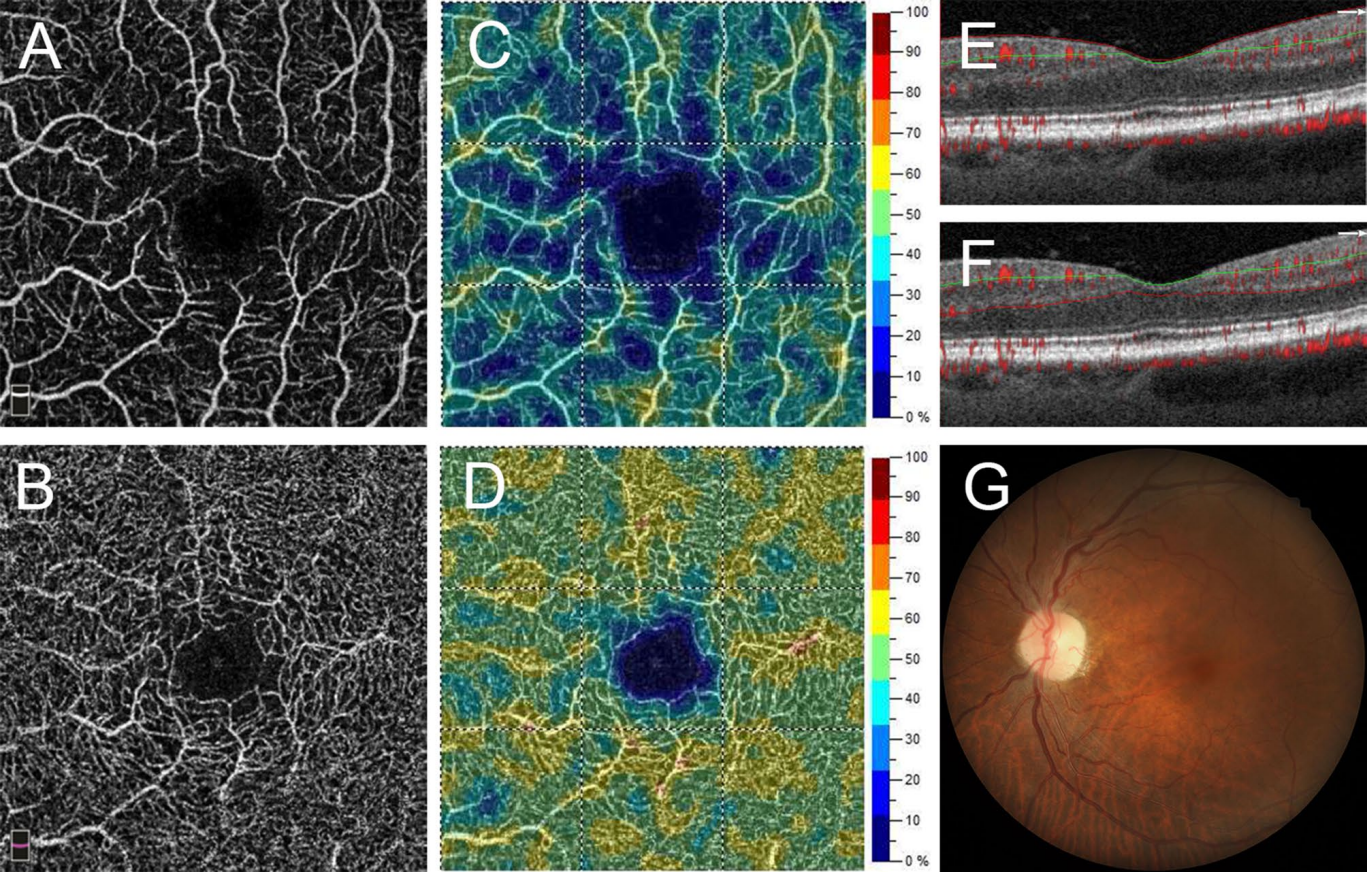
A representative patient with chronic hypertension with severe retinal microvascular rarefaction in the superficial vascular plexus (SVP) but relatively good vessel density in the deep vascular plexus (DVP). (**A**,**B**) Optical coherence tomography angiography (OCTA) images of the SVP and DVP. (**C**,**D**) are the vessel density maps of the SVP and DVP. (**E**) B-scan shows the segmentation boundaries of SVP and (**F**) boundaries of DVP. (**G**) Color fundus photo shows mild tortuous retinal vessels.

Figure 1 shows a representative patient with good hypertension control and normal microvasculature in both SVP and DVP. Two representative patients with poor SBP control and moderate SVP microvascular injury are shown in Fig. 2. Figure 3 demonstrates a patient with chronic hypertension with severe microvascular rarefaction in SVP but relative good vessel density in DVP.

## Discussion

Microvascular rarefaction in both SVP and DVP has been found in patients with CKD, but the role of hypertension is yet to be determined^12,13,19,20^. The effect of blood pressure on different layers of retinal microvasculature is controversial, and diverse results have been found in patients with hypertension^10,15–17^. Chua et al*.* measured capillary density within a circular region of 1 mm diameter centered on the fovea in a group of patients with uncomplicated hypertension^10^. They found that compared with patients with well-controlled BP, patients with poorly controlled BP showed a lower foveal capillary density at DVP. In another study, Ciloglu et al*.* demonstrated that compared with healthy controls or pregnant women without pre-eclampsia, those with pre-eclampsia had lower parafoveal DVP vessel density^17^. By contrast, Lee et al*.* found decreased parafoveal SVP vessel density in patients with chronic hypertension (≥ 10 years) or relieved hypertensive retinopathy^15^. Similarly, Hua et al*.* showed that compared with healthy controls, patients with a history of hypertension for 5–10 years or more than 10 years showed decreased parafoveal SVP vessel density^16^. Our study also suggested that BP control is significantly correlated with the microvascular injury in SVP. These discrepancies might be attributed to the different severity and chronicity of hypertension in different studies.

Chua et al*.* excluded patients with end-organ complications (such as significant coronary artery disease, strokes, and atrial fibrillation), and the mean eGFRs exceeded 90 mL/min/1.73 m^2^ in both groups in their study^10^. The “poorly” and “well” controlled BP groups were classified by a 24-h ambulatory BP measurement in their study. Similarly, pre-eclampsia in pregnant women is a temporary disorder in which their BP is usually carefully controlled and monitored by gynecologists after diagnosis. The intergroup difference in DVP vessel density in the above conditions might represent short-term vascular changes in response to BP elevation through autoregulation. Autoregulation is an important mechanism for maintaining a constant blood flow to the retina^21,22^. Retinal vasoconstriction can be readily observed in acute blood pressure elevation^21^. Improvement in retinal capillary rarefaction has also been reported after commencement of antihypertensive treatments in patients with hypertension^11^.

Patients in the studies by Lee et al*.* and Hua et al*.* had either a long-term history of hypertension, or end-organ damage (e.g. hypertensive retinopathy)^15,16^. Our study also enrolled patients with specific end-organ disease, i.e. CKD. The decreased SVP vessel density in these patients may represent an accumulated or persistent damage of retinal microvasculature owing to the hypertension. A rodent model demonstrated the failure of autoregulation and morphological changes in retinal vessels after long-term hypertension^21^. Combining all these evidences and our results, we speculate that, in patients with CKD, retinal microcirculation in SVP might be more susceptible to injuries caused by chronic hypertension than retinal microcirculation in DVP is. Further longitudinal studies are required to confirm this speculation. Some patients in this study had severe microvascular rarefaction in SVP but relative good vessel density in DVP (Fig. 3).

The SVP and DVP within the retina may respond differently to changes in systemic conditions. Histological studies have shown that there are three layers of retinal capillary over the macula: the superficial, middle, and deep capillary plexus^23^. Each of the three capillary plexuses has its own arteriolar and venous supply^24^, and may respond differently to physiological changes^22,25,26^. The SVP is composed of long, horizontal branching arterioles and venules originating from the superior and inferior arcades and interconnected by transverse capillaries^27^. The DVP is composed of capillary vortex, in which the capillaries converge toward an epicenter^27^. The resistance of blood flow may increase with the progressively decreased vessel diameter from the arterioles to the interconnecting capillaries in SVP. Elevated BP may cause vasoconstriction^21,22^. Chronic hypertension may also lead to atherosclerosis^1^, which makes the vessels narrower, further increasing the resistance of the vessels and dysfunction of autoregulation^10,19,28–30^. The increased resistance slows down the blood flow and may cause focal flow void area on OCTA^10^.

The maximum SBP and within-patient SD of SBP were *negatively* correlated with SVP vessel density, while *positively* correlated with SVP flow void area. Both vessel density and flow void area could be biomarkers for retinal vascular injury^8,15,16,31^. More severe vascular injury tends to present a larger flow void area and a lower vessel density. An inverse relationship between flow void area and vessel density in SVP was confirmed in this study (Pearson correlation coefficient − 0.389, p < 0.001). However, it is intriguing that average SBP and proportion of high SBP measurement correlated only with SVP flow void area, but not with SVP vessel density. This might imply that increased parafoveal focal flow void area, which represents focal vascular defects, in the SVP may be an early sign of microvascular injury in patients with CKD. It might be a more sensitive parameter than SVP vessel density, which represents more diffuse microvascular changes in the retina.

SBP seems more important than DBP is in causing SVP microvascular injuries in this study. This observation is consistent with the prior theory that SBP was associated with systemic vascular complications such as cardiovascular disease, cerebrovascular disease, progression of CKD, and mortality^32–34^. Controlling SBP may lower the risk of above complications^35–37^. However, DBP has recently attracted attention on its ability to predict cardiovascular outcome, especially in younger subjects^38,39^; its effects in retinal microvasculature should be further examined.

The BP parameters in the past 1 year, but not SBP/DBP at enrollment, correlated with microvascular parameters. This indicated that long-term BP control is more important than temporary BP elevation in causing microvascular changes in SVP. Furthermore, our study also showed that BP variability correlates better with SVP microvascular changes than with average BP. Visit-to-visit variability of BP is associated with the risk of cardiovascular diseases, cerebrovascular disease, progression of CKD and mortality^40–43^. Possible etiology may involve endothelial dysfunction, arterial stiffness and arterial remodeling^44–46^. Our study demonstrated BP variability’s potential influence on retinal microvasculature. Since retinal microvascular changes could be readily observed in vivo, they could be useful in future studies on the mechanism of vascular injury from BP variability.

There are various methods to measure visit-to-visit BP variation^40,47,48^. Prior studies showed that 5–6 BP readings could be adequate to identify the relationship between visit-to-visit BP variation and end-organ injury^40,42,47–49^. Most patients follow up regularly in 1–3 month intervals in our study. The average number of BP measurements for each patient is 8.7 in the past 1 year. This could provide enough data for evaluating the BP variability in this study.

Because human BP exhibits a circadian rhythm, ambulatory 24 h BP monitoring is considered a better assessor of BP variability and a better predictor of cardiovascular events than traditional office BP is^50^. However, due to the associated cost and discomfort, ambulatory monitoring is usually only performed during the initial diagnosis of hypertension or when out-of-office hypertension is suspected (e.g. non-dipping hypertension or masked hypertension)^50^. Although we did not have ambulatory BP data in this study, most of our patients visit the same clinic regularly and BPs were measured at similar time points during their visits. This may minimize the influence of circadian rhythm.

Our hospital generally used unattended automated office BP (AOBP)—which could be similar to daytime ambulatory BP and is more accurate than traditional office BP measurement^51^. Because of high patient flow in our hospital, our patients’ BP was measured once in each routine follow up visit, instead of 3 times. It has been shown that after appropriate resting, the first AOBP measurement could highly correlate with the mean of 3 AOBP measurements (r = 0.92)^47^. A recent study also showed that one BP measurement appears adequate for patients with normal initial BP values^52^. Because patients with initial BP higher than normal could benefit from multiple BP measurements^52^, our hospital does perform multiple BP measurements for patients with higher than normal BP upon the physician’s request. Our patients were also encouraged to perform home BP monitoring (HBPM) and submit the data during follow-up visits. HBPM is as accurate as ambulatory BP^51,53^, and may facilitate physician’s adjustment of antihypertensive medications.

DM was a covariate in our partial correlation models because it may cause retinal microvascular changes^8^, which could affect OCTA parameters in CKD patients^12^. Among CKD patients with DM, the HbA1c level was correlated with SVP vessel density. We repeated the statistical analysis in this subgroup by replacing DM with HbA1c as a covariate in the partial correlation models. The results were similar to those from the entire CKD group except that the correlation between average SBP and SVP flow void area had become insignificant (p = 0.253). The maximum SBP and within-patient SD of SBP were correlated with SVP vessel density (p = 0.008 and 0.006) and SVP flow void area (p = 0.003 and < 0.001). This may support that BP variability and blood sugar control are independent factors for microvascular injuries in this subgroup of patients.

This study has several limitations, one of which is its small sample size. Subgroup analysis (such as different stages or different etiologies of CKD) was difficult in this study owing to the small number of patients. Moreover, the duration of CKD and of hypertension had no significant correlation with OCTA parameters. However, the duration may be inaccurate because these diseases are typically diagnosed with an unspecified delay after onset. The effect of different classes of antihypertensive medications was not evaluated in this study. The variation in the classes and the dosage of medications used make statistical analyses difficult for the small sample size of this study. Finally, ambulatory 24 h BP monitoring data from the CKD group and long-term BP data from the control group were not available.

In summary, our study demonstrated that the long-term BP parameters correlated with parafoveal SVP vessel density and parafoveal SVP flow void area on OCTA in CKD patients. The SBP variability may play an important role in the SVP microvascular injury. Further longitudinal studies are warranted to confirm whether retinal microvascular parameters on OCTA can be potential biomarkers to help monitor long-term BP control in CKD patients. Animal models would also help illustrate the possible mechanisms of these retinal vascular injuries.

## Methods

We conducted a prospective case–control study between August 2017 and July 2019 in the Department of Nephrology and the Department of Ophthalmology in our hospital. This study was approved by the Chang Gung Memorial Hospital Institutional Review Board (IRB No.: 201602022B0 and 201702074A3) and followed the tenets of the Declaration of Helsinki.

### Participants

CKD was defined as (1) abnormalities of kidney structure or function that present for more than 3 months and (2) eGFR < 60 mL/min/1.73 m^2^ for more than 3 months^54^. Using the Chronic Kidney Disease Epidemiology Collaboration equation, eGFR was calculated from serum Cr^55^. Patients with CKD who were regularly followed up in the Nephrology Department for ≥ 1 year were invited to participate in this study. CKD was classified according to the eGFR values into stage 3 (30–59 mL/min/1.73 m^2^), stage 4 (15–29 mL/min/1.73 m^2^), and stage 5 (< 15 mL/min/1.73 m^2^). The inclusion criteria for the CKD group were patients (1) with CKD stages 3–5 (including end-stage renal disease); (2) aged ≥ 21 years; and (3) with no visual symptoms. Controls were age-matched healthy subjects without retinal disease in a 4:1 ratio. Patients with (1) significant ocular media opacity; (2) inadequate quality of spectral-domain optical coherence tomography (SD-OCT) or OCTA image (signal strength index < 50, quality score < 6, or presence of significant artifact); (3) pregnancy; (4) axial length ≥ 26 mm; (5) blood pressure measurements undertaken < 3 times in the past 1 year in the CKD group; or (6) presence of any diabetic retinopathy were excluded. Written informed consent was provided by each participant. If both eyes were illegible, the eye with better OCTA image quality was chosen for statistical analysis.

### Blood pressure measurement

All patients underwent BP measurement at each visit in the Nephrology Department, as well as on the date of enrollment. An unattended automated office BP (AOBP) was measured in a BP Suite of our hospital. Patients sat with their backs supported for at least 15 min. SBP and DBP were then measured using an automatic blood pressure monitor (FT-500R; Jawon Medical Co. Ltd., Korea). The data would then be automatically recorded into the patients’ electronic medical records. Patients with BP above normal may receive repeat BP measurements at the physician’s discretion.

The average SBP/DBP in 1 year were calculated by averaging all available blood pressure measurements in the past 1 year. The maximum SBP/DBP indicated the highest recorded SBP/DBP in the past 1 year. The within-patient SD of SBP/DBP, defined as the SD of all BP measurements of individual patients within the past 1 year, was used to evaluate the within-patient blood pressure fluctuation. The proportion of high SBP measurement in each CKD patient was defined as the number of measurements with SBP > 140 mmHg/total number of BP measurements.

### Ocular exams

A complete ocular exam was performed for each patient on the date of enrollment. BCVA was measured using a Snellen chart and converted to the logarithm of the minimum angle of resolution (logMAR) for statistical analysis. Intraocular pressure and axial length were measured using a non-contact tonometer (NT-3000; NIDEK, Gamagori, Aichi, Japan) and an IOLMaster (Carl Zeiss Meditec, Jena, Germany), respectively. SD-OCT and OCTA images were acquired using AngioVue (RTVue XR Avanti; Optovue, Inc., Fremont, CA, USA). Retinal microvascular parameters including parafoveal SVP vessel density, parafoveal DVP vessel density, foveal avascular zone, and parafoveal SVP flow void area were determined from OCTA images.

### OCTA image analysis

The acquisition and analysis of OCTA images were similar to our prior report^12^. In brief, the OCTA image of each eye was acquired through a 304 × 304 A-scan covering an area of 3 × 3 mm^2^ centered on the fovea in the macular region. The AngioVue software (version: A201710151) automatically segmented the vascular area into four layers, i.e., superficial, deep, outer retina, and choroidal. The SVP included the vasculature between the internal limiting membrane and 10 μm above the inner plexiform layer; the DVP included the vasculature between 10 μm above the inner plexiform layer and 10 μm below the outer plexiform layer^12^. Vessel density was defined as the percentage area occupied by all the large and small vessels within the region of interest. The foveal region is a circle measuring 1 mm in diameter, and the parafoveal region is a 1-mm wide circular annulus. Foveal avascular zone size, parafoveal SVP vessel density, and parafoveal DVP vessel density were automatically calculated by the AngioVue software.

These OCTA parameters had good reliability and reproducibility^56,57^. We conducted an in-house validation of the OCTA vessel density measurements by using the data from 20 eyes without retinopathy. The mean intra-visit coefficients of variation (CV) was 1.7% (SD 1.9%) for SVP and 1.5% (SD 1.6%) for DVP; while the inter-visit CV was 3.3% (SD 3.8%) and 3.1% (SD 3.0%), respectively.

### Calculation of parafoveal SVP flow void area

**Figure 4:**
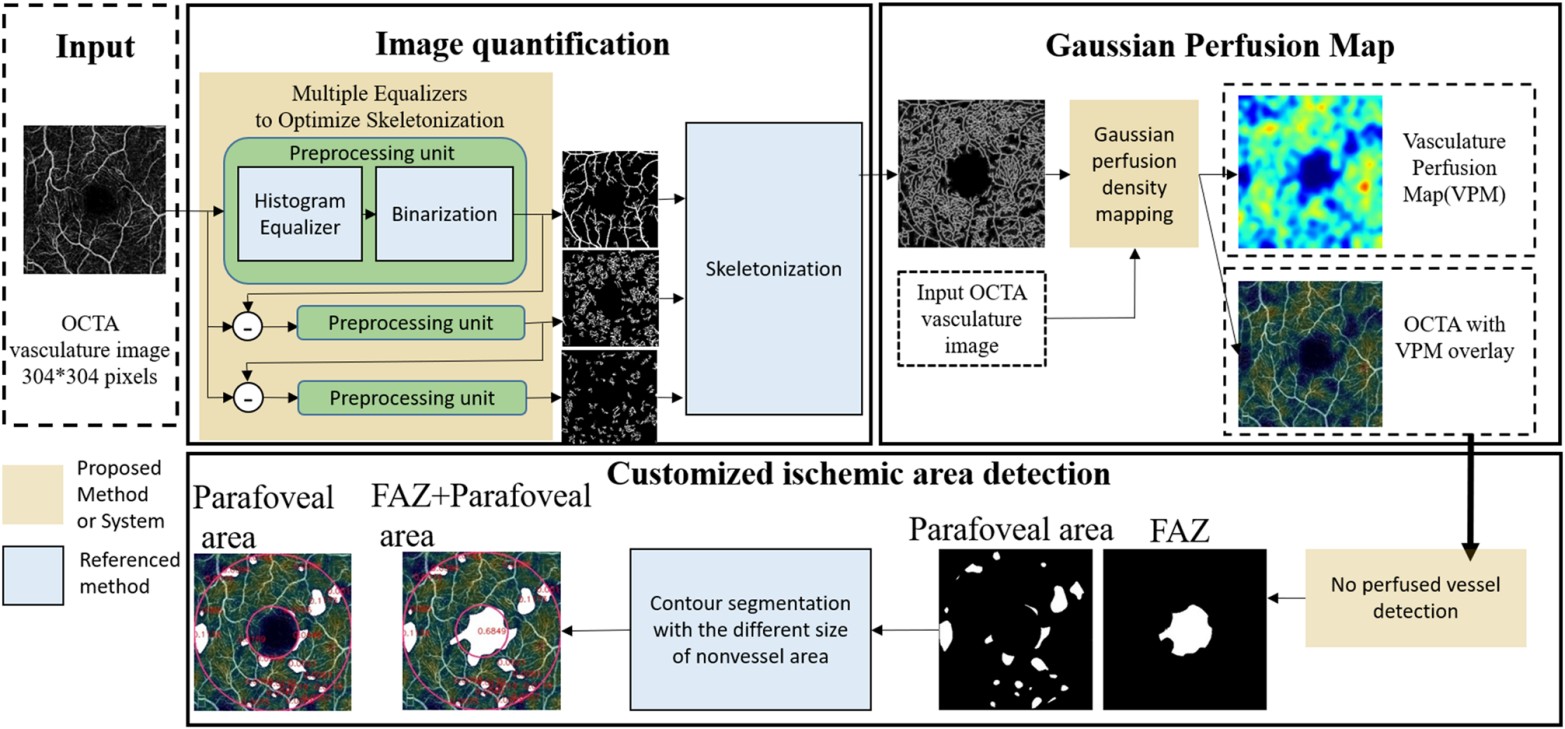
Flowchart of the proposed automatic OCTA image analysis algorithm.

The parafoveal SVP flow void area in each eye was determined from a superficial OCTA image. It was analyzed automatically by Python codes using three developed systems, including (1) image quantification, which applies three histogram equalizers to optimize the skeleton method, (2) generation of the Gaussian density map system, and (3) ischemic area detection (Fig. 4).

In the process of OCTA image quantification, OpenCV color histogram equalizers^58^ are applied to separate the obvious large vessel and the obscure small vessel. Given a grayscale image *x*, after applying the equalizer the pixel intensity *x*(*i*, *j*) is converted to *h*(*x*(*i*, *j*)), where

1 $$h\left(v\right)= round \left(\frac{255*cdf\left(v\right)}{MN}\right)$$

*cdf*(*v*) is the cumulative distribution function (i.e., the number of pixels whose intensity is no larger than *v*), and *MN* is the size of the image.

According to the statistics of the vessel color distribution, a pixel value around 185 is chosen as the threshold for binarization. After performing the color histogram and binarization three times, the vessels with a variety of sizes can be well extracted. Further, according to previous studies^59–61^, to calculate the perfusion area, we apply Zhang’s skeletonized method^36^ to quantify the density vessel. Second, the Gaussian Blur filter in OpenCV with a block size of 35 is used to define the perfused area rather than using the color-coded based method^60^, because the Gaussian Blur filter facilitates representation of the perfused area of OCTA images. The Gaussian Blur filter *G*(*i*, *j*) is given as

2 $$G(i,j)= \alpha *{e}^{\frac{-({i-(\frac{L-1}{2}))}^{2}+({j-(\frac{L-1}{2}))}^{2}}{2{\sigma }^{2}}}$$

3 $$\sigma =0.3*((L-1)*0.5-1)+0.8$$

where *L* is the block size, *i*, *j* = 0, 1, …, *L* − 1, and *α* is the scale factor to make $\sum_{i}\sum_{j}G\left(i,j\right)=1$.

Third, after generating a clear perfusion map, the ischemic areas are identified. The non-perfused vessel detection algorithm is applied to identify low-pixel-value regions, which are the regions with less blood vessels, from the vasculature Perfusion Map. Finally, the famous contour detection method^37^ is adopted to calculate the areas of non-vessel regions and determine the sizes of the foveal avascular zone over the parafoveal area.

### Statistical analysis

The demographic data and clinical characteristics between the CKD and control groups were compared using Pearson's chi-squared test for categorical variables and the independent sample *t*-test for continuous variables. The difference between the start and the end of the study was evaluated by comparing the first and last BP measurement by paired sample *t*-test. Partial correlation was used to determine whether BP parameters, duration of CKD, and duration of hypertension correlated with parafoveal retinal microvascular parameters among patients with CKD after controlling for age, sex, DM, axial length, and intraocular pressure. Among patients with CKD and DM, partial correlation was used to determine the relationship between recent HbA1c level and parafoveal retinal microvascular parameters after controlling for age, sex, axial length, and intraocular pressure. A two-tailed P value < 0.05 was considered statistically significant. All data were analyzed using SPSS version 17.0 (SPSS Inc., Chicago, IL, USA).

## Data Availability

The data is not publicly available due to the involvement of human participants and the terms of consent to which the participants had agreed. Nevertheless, upon reasonable request, the data is available from the corresponding author after obtaining Chang Gung Memorial Hospital Institutional Review Board approval.
